# Supplementary material for: Generation of Non-Nucleotide CD73 Inhibitors Using a Molecular Docking and 3D-QSAR Approach
Source: Int J Mol Sci. 2021 Nov 25;22(23):12745. doi: 10.3390/ijms222312745 (PMC8657903; doi:10.3390/ijms222312745)
Supplement: Supplementary file 1 [file ijms-22-12745-s001.zip › ijms-1476285-supplementary.pdf]

Research Article

Generation of non-nucleotide CD73 inhibitors using a molecular docking and 3D-QSAR approach

Swapnil P. Bhujbal[1,2] and Jung-Mi Hah[1,2*]

[1] College of Pharmacy, Hanyang University, Ansan 426-791, Korea; swapnil18@hanyang.ac.kr (S.P.B.);
[2] Institute of Pharmaceutical Science and Technology, Hanyang University, Ansan 426-791, Korea; jhah@hanyang.ac.kr;
* Correspondence: jhah@hanyang.ac.kr; Tel.: +82-31-400-5803

Supplementary Material

Table S1: The chemical structures of the selected non-nucleotide small molecule inhibitors with their IC_{50} (μM) values.

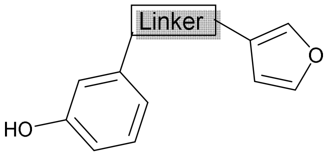

Compounds 1 to 4

Compound	Linker	IC_{50} (μM)
1	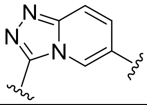	15.6
2	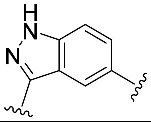	1000
3	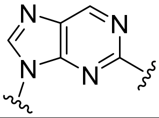	99
4	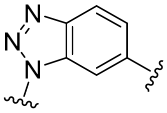	10

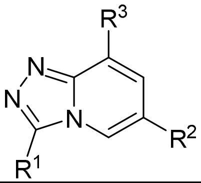

Compounds 5 to 17				
Compound	R^1	R^2	R^3	IC_{50} (μM)
5*	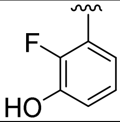	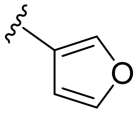	H	450
6	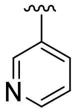	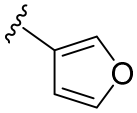	H	97.3
7	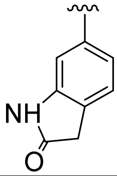	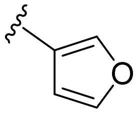	H	148
8*	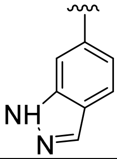	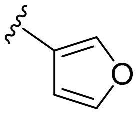	H	5.2
9*	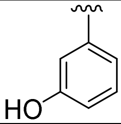	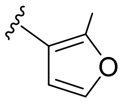	H	12.4
10	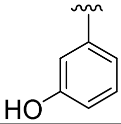	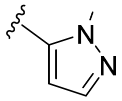	H	13.7
11*	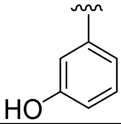	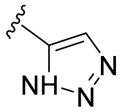	H	178.5
12	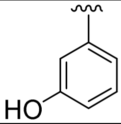	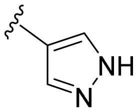	H	68.1
13*	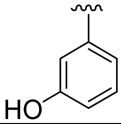	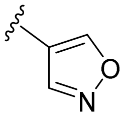	H	191
14	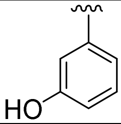	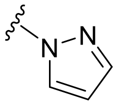	H	121
15	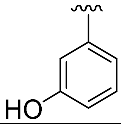	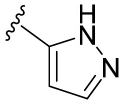	H	54.4
16	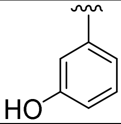	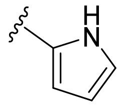	H	47.0

17	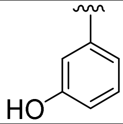	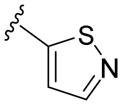	H	9.0
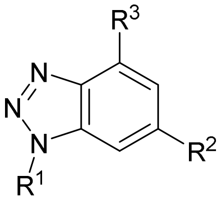 Compounds 18 to 56				
18	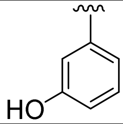	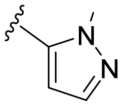	H	22.3
19	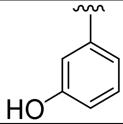	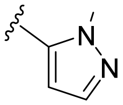	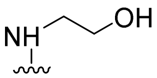	49.0
20	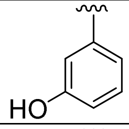	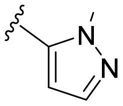	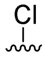	1.7
21	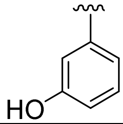	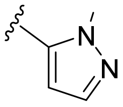	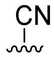	12.0
22*	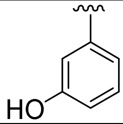	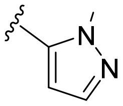	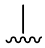	5.1
23	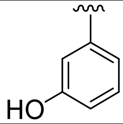	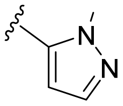	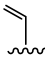	14.2
24	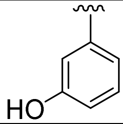	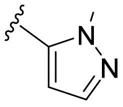	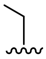	140
25*	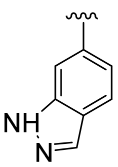	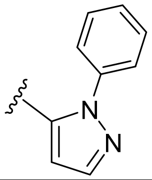	H	9.2
26	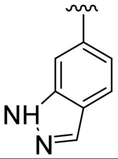		H	0.306

27*	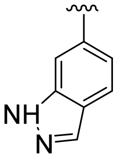	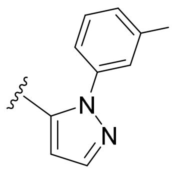	F	0.333
28	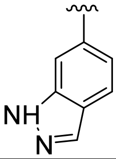		Cl	0.220
29	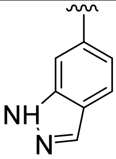	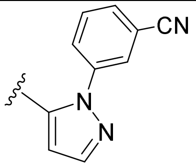	H	2.85
30	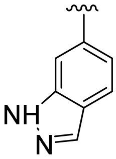	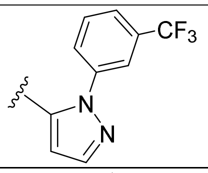	H	0.331
31	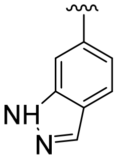	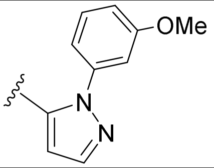	H	0.646
32	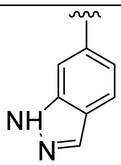	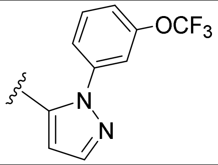	H	0.408
33*	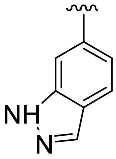	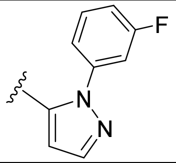	H	2.39
34	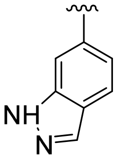	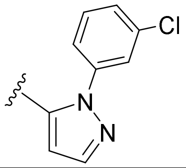	H	0.360
35	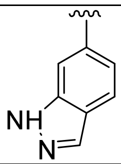	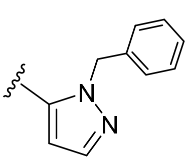	H	1.08
36	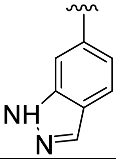		F	0.210

37	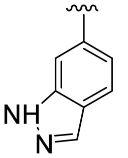		Cl	0.141
38*	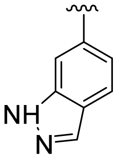	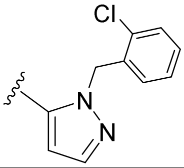	H	0.142
39	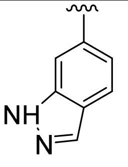	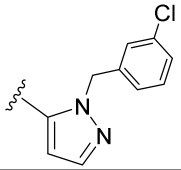	H	1.03
40	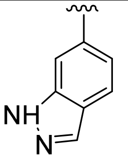	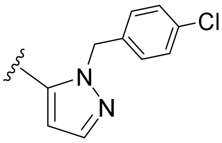	H	0.065
41	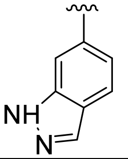	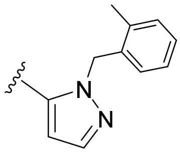	H	0.224
42*	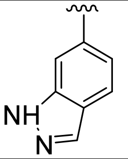	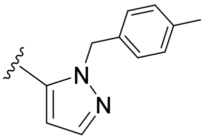	H	0.078
43*	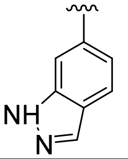	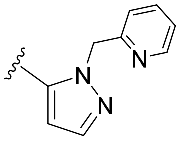	H	0.269
44	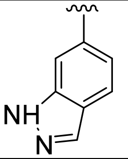	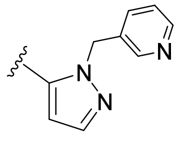	H	3.69
45	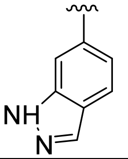	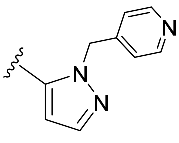	H	0.651
46*	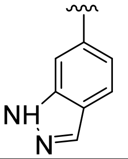	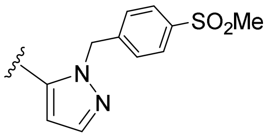	H	5.35

47	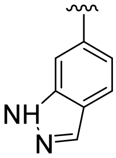	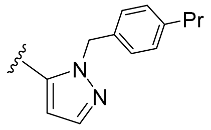	H	0.594
48	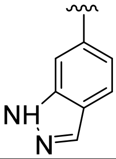		H	0.021
49	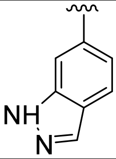	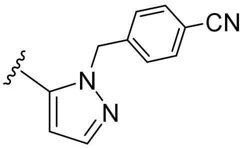	F	0.030
50	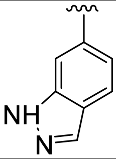		Cl	0.022
51	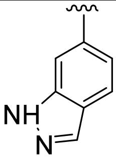	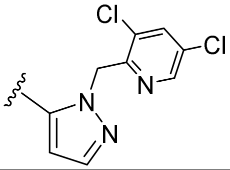	H	0.480
52	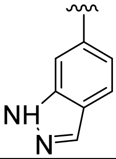	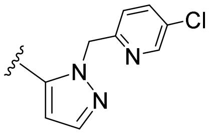	H	0.162
53	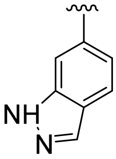	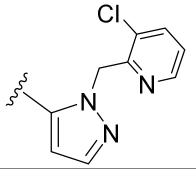	H	6.75
54*	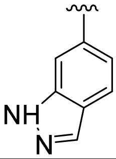	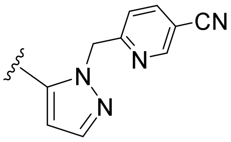	H	0.057
55	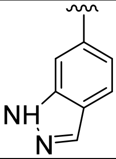	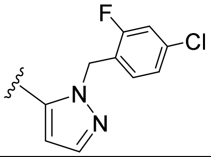	H	0.054
56	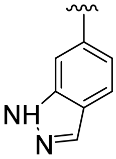	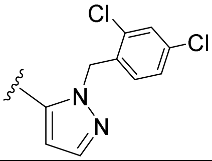	H	0.045

*denotes test set compounds in Table S1.

Table S2: The structures and the predicted pIC_{50} values of new designed CD73 inhibitors.

Compound	R^1	R^2	R^3	R^4	Predicted pIC_{50}
**R^1 Selected**					
D1	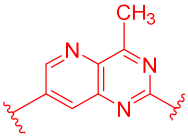	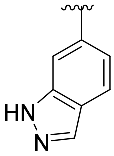	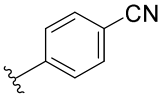	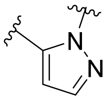	7.038
D2	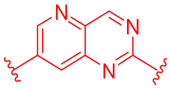				6.374
D3	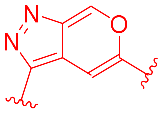			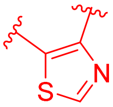	7.531
**R^2 Selected**					
D4	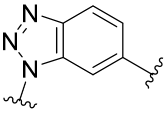	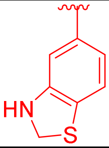	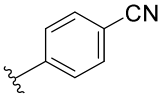	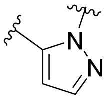	7.59
D5		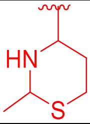			7.603
D6		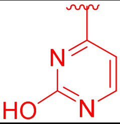			7.54
D7		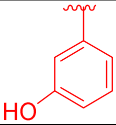			7.644
**R^3 Selected**					
D8	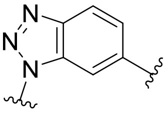	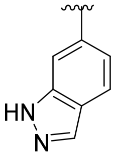	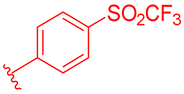	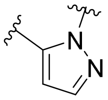	7.645
D9			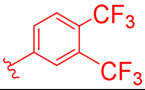		7.621
D10			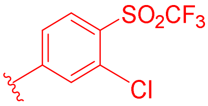		8.013
D11			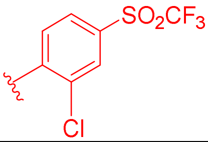		8.056

D12			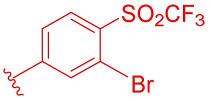		8.134
D13			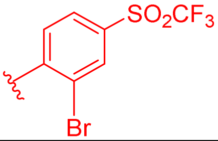		8.163
**R⁴ Selected**					
D14	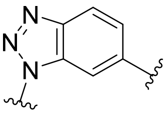	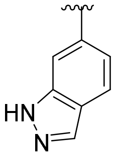	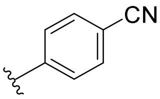	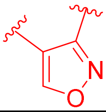	7.769
D15				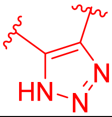	7.769
D16				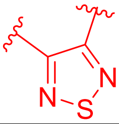	7.74
D17				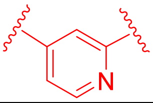	7.649
**Compounds with two or more modified substituents**					
D18	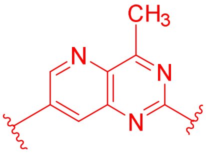	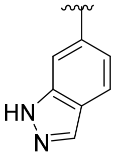	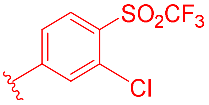	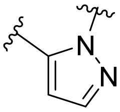	7.693
D19			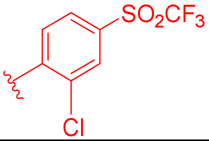		7.609
D20			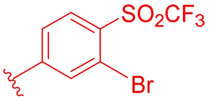		7.839
D21			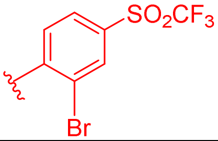		7.73
D22	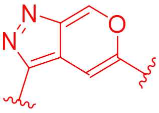		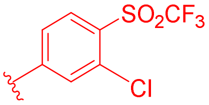		7.959
D23			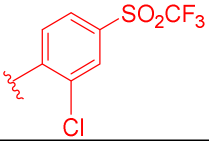		7.855
D24			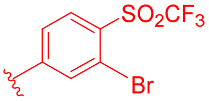		8.088

D25			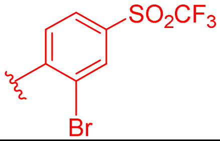		7.976
D26			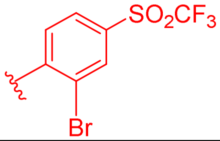	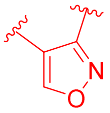	8.098
D27				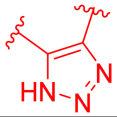	7.703
D28			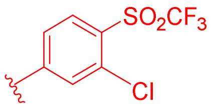	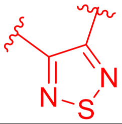	7.872
D29				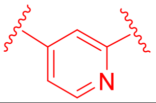	7.844
D30			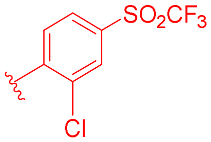	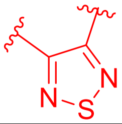	7.815
D31				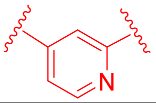	7.791
D32	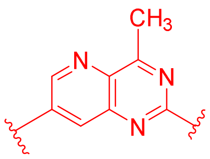	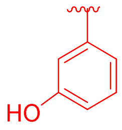		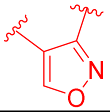	7.879
D33			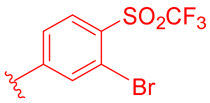	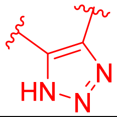	8.047
D34				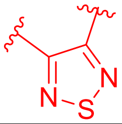	8.018
D35			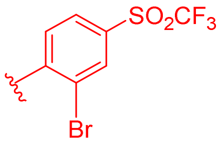	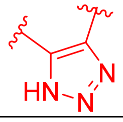	7.929
D36				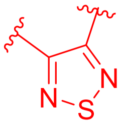	7.911
D37				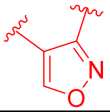	8.092

D38	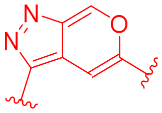		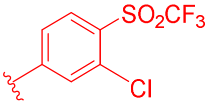	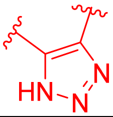	8.107
D39				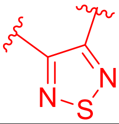	8.067
D40				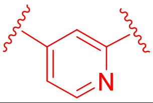	7.889
D41			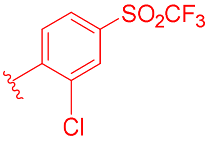	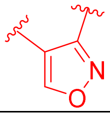	7.985
D42				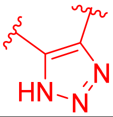	7.996
D43				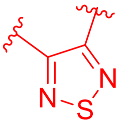	7.956
D44				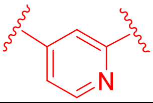	7.785
D45			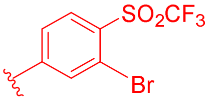	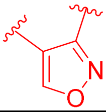	8.236
D46				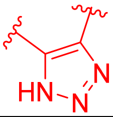	8.251
D47				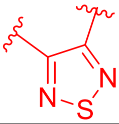	8.211
D48				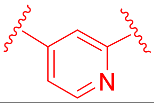	8.052
D49			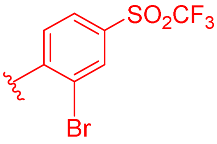	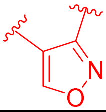	8.095
D50				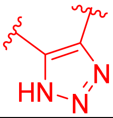	8.112
D51				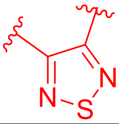	8.071

D52				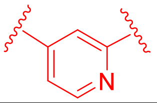	7.913

Table S3: CoMSIA models developed using different combinations of fields.

**CoMSIA**	***q*²**	**ONC**	**SEP**	***r*²**	**SEE**	**F value**
S	0.711	2	0.682	0.746	0.639	77.786
E	0.753	2	0.630	0.815	0.545	117.069
H	0.731	2	0.657	0.791	0.579	100.487
A	0.768	5	0.628	0.882	0.448	74.836
D	0.618	1	0.776	0.654	0.739	102.085
SE	0.753	3	0.636	0.829	0.529	84.203
EH	0.766	3	0.620	0.857	0.484	103.825
EA	0.743	3	0.648	0.864	0.471	110.569
ED	0.741	3	0.651	0.824	0.537	81.268
SH	0.730	1	0.652	0.758	0.618	169.169
SA	0.750	5	0.652	0.890	0.432	81.112
SD	0.681	4	0.730	0.779	0.607	45.009
HA	0.775	5	0.619	0.927	0.353	126.956
HD	0.720	3	0.677	0.830	0.527	84.82
SEH	0.760	3	0.627	0.844	0.505	93.911

SEA	0.740	3	0.652	0.855	0.488	102.88
SED	0.735	3	0.659	0.832	0.525	85.39
EHA	0.762	3	0.624	0.876	0.450	122.803
EHD	0.765	3	0.620	0.866	0.469	111.870
SHA	0.772	5	0.623	0.931	0.343	134.406
SHD	0.720	1	0.664	0.750	0.628	161.60
EAD	0.734	4	0.666	0.892	0.424	105.798
HAD	0.765	6	0.639	0.939	0.323	125.36
SEHD	0.759	3	0.629	0.862	0.475	108.686
SEHA	0.757	4	0.637	0.907	0.394	124.152
SEAD	0.730	3	0.665	0.866	0.469	111.926
EHAD	0.764	4	0.627	0.916	0.375	138.96
**SHAD**	**0.760**	**6**	**0.646**	**0.943**	**0.314**	**135.938**
SEHAD	0.755	3	0.634	0.881	0.442	127.80

Table S4: The predicted pIC_{50} and residual values for both CoMFA and CoMSIA (SHAD) models.

Compound	Actual pIC_{50}	CoMFA		CoMSIA (SHAD)	
		Predicted pIC_{50}	Residual	Predicted pIC_{50}	Residual
01	4.807	4.381	-0.426	4.420	-0.387
02	3.000	3.067	0.067	3.145	0.145
03	4.004	4.227	0.223	4.002	-0.002
04	5.000	4.967	-0.033	4.678	-0.322
05	3.347	4.401	1.054	4.431	1.084
06	4.012	4.186	0.174	4.135	0.123
07	3.830	4.046	0.216	3.737	-0.093
08	5.284	4.284	-1.000	5.178	-0.106
09	4.907	4.685	-0.222	4.744	-0.163

10	4.863	4.559	-0.304	4.743	-0.120
11	3.748	4.184	0.436	4.680	0.932
12	4.167	4.083	-0.084	4.331	0.164
13	3.719	4.196	0.477	4.315	0.596
14	3.917	4.369	0.452	4.303	0.386
15	4.264	4.304	0.040	4.345	0.081
16	4.328	4.283	-0.045	4.000	-0.328
17	5.046	4.963	-0.083	4.745	-0.301
18	4.652	4.864	0.212	5.084	0.432
19	4.310	4.031	-0.279	4.637	0.327
20	5.770	5.183	-0.587	5.386	-0.384
21	4.921	5.336	0.415	5.090	0.169
22	5.292	4.466	-0.826	5.066	-0.226
23	4.848	4.755	-0.093	5.031	0.183
24	3.854	3.949	0.095	3.984	0.130
25	5.036	5.970	0.934	6.098	1.062
26	6.514	6.464	-0.050	6.314	-0.200
27	6.478	6.690	0.212	6.417	-0.061
28	6.658	6.747	0.089	6.611	-0.047
29	5.545	5.971	0.426	5.781	0.236
30	6.480	6.370	-0.110	6.510	0.030
31	6.190	6.286	0.096	6.015	-0.175
32	6.389	6.301	-0.088	6.463	0.074
33	5.622	5.922	0.300	6.176	0.554
34	6.444	6.133	-0.311	6.351	-0.093
35	5.967	6.229	0.262	6.466	0.499
36	6.678	6.503	-0.175	6.570	-0.108
37	6.851	6.399	-0.452	6.753	-0.098
38	6.848	6.203	-0.645	6.575	-0.273
39	5.987	6.254	0.267	6.374	0.387
40	7.187	6.969	-0.218	7.099	-0.088
41	6.650	6.269	-0.381	6.663	0.013
42	7.108	6.430	-0.678	6.801	-0.307
43	6.570	5.721	-0.849	6.144	-0.426
44	5.433	5.537	0.104	5.117	-0.316
45	6.186	5.977	-0.209	5.887	-0.299
46	5.272	5.916	0.644	5.777	0.505
47	6.226	6.320	0.094	6.653	0.427
48	7.678	7.735	0.057	7.568	-0.110
49	7.523	8.009	0.486	7.672	0.149
50	7.658	7.910	0.252	7.855	0.197
51	6.319	6.495	0.176	6.205	-0.114
52	6.790	6.524	-0.266	6.094	-0.696
53	5.171	5.860	0.689	5.583	0.412
54	7.244	6.763	-0.481	6.407	-0.837

55	7.268	6.967	-0.301	7.123	-0.145
56	7.347	6.945	-0.402	7.208	-0.139

Table S5: In silico ADME prediction and synthetic accessibility values of designed CD73 inhibitors.

Compound	Lipophilicity	Water Solubility		Pharmacokinetics		Synthetic Accessibility	Druglikeness
	Log $P_{o/w}$ (XLOGP3)	Log *S* (*ESOL)*	Class	GI absorption	Toxicity (AMES) Categorical (Yes/No)		Lipinski rule
D1	3.54	-5.20	Moderately soluble	High	No	3.28	Yes; 0 violation
D2	4.69	-6.25	Poorly soluble	Low	No	3.98	Yes; 1 violation
D3	3.63	-5.23	Moderately soluble	Low	No	3.92	Yes; 0 violation
D4	4.43	-5.67	Moderately soluble	High	No	3.55	Yes; 0 violation
D5	3.35	-4.76	Moderately soluble	High	No	4.39	Yes; 0 violation
D6	2.65	-4.33	Moderately soluble	High	No	33.28	Yes; 0 violation
D7	3.70	-4.98	Moderately soluble	High	Yes	3.11	Yes; 0 violation
D8	4.42	-6.05	Poorly soluble	Low	No	3.38	Yes; 1 violation
D9	5.74	-6.89	Poorly soluble	Low	No	3.43	No; 2 violations
D10	5.05	-6.65	Poorly soluble	Low	No	3.42	Yes; 1 violation
D11	5.05	-6.65	Poorly soluble	Low	No	3.45	Yes; 1 violation
D12	5.11	-6.96	Poorly soluble	Low	No	3.50	Yes; 1 violation
D13	5.11	-6.96	Poorly soluble	Low	No	3.49	Yes; 1 violation
D14	4.15	-5.45	Moderately soluble	High	No	3.49	Yes; 0 violation
D15	3.49	-5.03	Moderately soluble	High	No	3.46	Yes; 0 violation
D16	4.19	-5.58	Moderately soluble	Low	No	3.40	Yes; 0 violation
D17	4.64	-5.82	Moderately soluble	High	Yes	3.26	Yes; 0 violation
D18	4.90	-6.71	Poorly soluble	Low	No	3.51	Yes; 1 violation
D19	4.90	-6.71	Poorly soluble	Low	No	3.54	Yes; 1 violation
D20	4.96	-7.02	Poorly soluble	Low	No	3.58	Yes; 1 violation

D21	4.96	-7.02	Poorly soluble	Low	No	3.58	Yes; 1 violation
D22	3.90	-5.93	Moderately soluble	Low	No	3.88	Yes; 1 violation
D23	3.90	-5.93	Moderately soluble	Low	No	3.88	Yes; 1 violation
D24	3.97	-6.25	Poorly soluble	Low	No	3.93	Yes; 1 violation
D25	3.97	-6.25	Poorly soluble	Low	No	3.92	Yes; 1 violation
D26	4.44	-6.37	Poorly soluble	Low	No	4.09	Yes; 1 violation
D27	4.71	-6.41	Poorly soluble	Low	No	3.70	Yes; 1 violation
D28	5.40	-6.96	Poorly soluble	Low	No	3.61	Yes; 1 violation
D29	5.85	-7.20	Poorly soluble	Low	No	3.56	Yes; 1 violation
D30	5.40	-6.96	Poorly soluble	Low	No	3.64	Yes; 1 violation
D31	5.85	-7.20	Poorly soluble	Low	No	3.55	Yes; 1 violation
D32	5.43	-7.14	Poorly soluble	Low	No	3.81	Yes; 1 violation
D33	4.77	-6.73	Poorly soluble	Low	No	3.75	Yes; 1 violation
D34	5.47	-7.27	Poorly soluble	Low	No	3.68	Yes; 1 violation
D35	4.77	-6.73	Poorly soluble	Low	No	3.76	Yes; 1 violation
D36	5.47	-7.27	Poorly soluble	Low	No	3.70	Yes; 1 violation
D37	4.38	-6.06	Poorly soluble	Low	No	4.07	Yes; 1 violation
D38	3.72	-5.64	Moderately soluble	Low	No	3.99	Yes; 1 violation
D39	4.41	-6.18	Poorly soluble	Low	No	3.99	Yes; 1 violation
D40	4.86	-6.43	Poorly soluble	Low	No	3.92	Yes; 1 violation
D41	4.38	-6.06	Poorly soluble	Low	No	4.06	Yes; 1 violation
D42	3.72	-5.64	Moderately soluble	Low	No	4.00	Yes; 1 violation
D43	4.41	-6.18	Poorly soluble	Low	No	3.99	Yes; 1 violation
D44	4.86	-6.43	Poorly soluble	Low	No	3.90	Yes; 1 violation
D45	4.44	-6.37	Poorly soluble	Low	No	4.10	Yes; 1 violation

D46	3.78	-5.96	Moderately soluble	Low	No	4.02	Yes; 1 violation
D47	4.48	-6.50	Poorly soluble	Low	No	4.02	Yes; 1 violation
D48	4.93	-6.75	Poorly soluble	Low	No	3.96	Yes; 1 violation
D49	4.44	-637	Poorly soluble	Low	No	4.09	Yes; 1 violation
D50	3.78	-5.96	Moderately soluble	Low	No	4.01	Yes; 1 violation
D51	4.48	-6.50	Poorly soluble	Low	No	4.03	Yes; 1 violation
D52	4.93	-6.75	Poorly soluble	Low	No	3.96	Yes; 1 violation

Where, Log $P_{o/w}$: partition coefficient between n-octanol and water; Log *S* (ESOL): decimal logarithm of the molar solubility in water.

Table S6: The docking score and various interactions of all docked designed CD73 inhibitors.

**Compound**	**Docking Score**	**H-Bond interactions**	**Pi-pi or pi-cation interactions**	**Halogen bond interaction**
D1	-8.621	Asp506	Phe417, Phe500, Arg354, Arg395	-
D2	-8.466	Asn390, Asp506	Phe417, Phe500, Arg354	-
D3	-9.682	Asp506	Phe417, Phe500, Arg354, Arg395	-
D4	-10.061	Asn390, Asp506	Phe417, Phe500, Arg354, Arg395	-
D5	-8.686	Asn390, Gly392	Phe417, Phe500	-
D6	-8.722	Asn390, Asp506	Phe417, Phe500, Arg395	-
D7	-9.639	Asn390, Asp506	Phe417, Phe500, Arg395	-
D8	-9.163	Asn390, Asp506	Phe417, Phe500, Arg354, Arg395	-
D9	-9.180	Asn390, Asp506	Phe417, Phe500, Arg354, Arg395	-
D10	-8.946	Asn390, Asp506	Phe417, Phe500, Arg354, Arg395	-
D11	-9.099	Asn390, Asp506	Phe417, Phe500, Arg354, Arg395	Asn503
D12	-9.389	Asn390, Asp506	Phe417, Phe500, Arg354, Arg395	-
D13	-9.708	Asn390, Asp506	Phe417, Phe500, Arg354, Arg395	Asn499, Asn503
D14	-9.603	Asn390, Asp506	Phe417, Phe500, Arg354, Arg395	-
D15	-8.936	Asn390, Asp506	Phe417, Phe500, Arg354, Arg395	-

D16	-9.721	Asn390, Asp506	Phe417, Phe500, Arg354, Arg395	-
D17	-9.122	Asn390, Asp506	Phe417, Phe500, Arg354, Arg395	-
D18	-9.234	Asp506	Phe417, Phe500, Arg354, Arg395	Asn186
D19	-9.339	Asp506	Phe417, Phe500, Arg354, Arg395	-
D20	-9.323	Asp506	Phe417, Phe500, Arg354, Arg395	Asn186
D21	-9.545	Asp506	Phe417, Phe500, Arg354, Arg395	-
D22	-9.282	Asn390, Asp506	Phe500, Arg354, Arg395	Asn503
D23	-9.678	Asn390, Asp506	Phe417, Phe500, Arg354, Arg395	Asn499, Asn503
D24	-9.027	Asn390, Asp506	Phe500, Arg354, Arg395	-
D25	-9.547	Asp506	Phe500, Arg354, Arg395	Asn499, Asn503
D26	-8.459	Asn390, Asp506	Phe417, Phe500, Arg395	-
D27	-8.490	Asn390, Asp506	Phe417, Phe500, Arg354	Asn186
D28	-8.924	Asp506	Phe417, Phe500, Arg395	Asn186
D29	-9.017	Asn390, Asp506	Phe417, Phe500, Arg395	-
D30	-8.984	Asn390, Asp506, Gly447	Phe417, Phe500, Arg354	Asn499, Asn503
D31	-8.898	Asp506	Phe417, Phe500, Arg395	-
D32	-8.938	Asp506	Phe417, Phe500, Arg395	Asn186
D33	-8.969	Asp506	Phe417, Phe500, Arg395	-
D34	-9.260	Asn186, Asp506	Phe417, Phe500, Arg354, Arg395	-
D35	-8.478	Asp506	Phe417, Phe500, Arg354, Arg395	-
D36	-9.092	Asn390, Asp506, Gly447	Phe417, Phe500, Arg354	Asn499, Asn503
D37	-8.909	Asn390, Asp506	Phe417, Phe500, Arg395	Asn503
D38	-8.658	Asn390, Asp506	Phe417, Phe500, Arg395	Asn503
D39	-8.726	Asn390, Asp506	Phe500, Arg395	-
D40	-8.913	Asp506	Phe500, Arg395	-
D41	-8.763	Asn390, Asp506	Phe417, Phe500, Arg395	Asn499, Asn503

D42	-8.874	Asn390, Asp506	Phe417, Phe500, Arg395	-
D43	-8.944	Asp506	Phe500, Arg395	Asn499, Asn503
D44	-8.694	Asp506	Phe500, Arg395	Asn503
D45	-8.328	Asn390, Asp506	Phe500, Arg395	Asn186
D46	-8.278	Asn390, Asp506	Phe417, Phe500, Arg395	Asn186
D47	-8.654	Asn390, Asp506	Phe500, Arg395	Asn503
D48	-8.667	Asp506	Phe500, Arg395	-
D49	-8.459	Asn390	Phe417, Phe500, Arg395	-
D50	-8.864	Asn390, Asp506, Asp121	Phe417, Phe500, Arg395	-
D51	-8.322	Asn390, Asp506	Phe417, Phe500, Arg395	Asn499, Asn503
D52	-9.135	Asp506	Phe500, Arg395	Asn503

Table S7: SMILES codes for the CD73 Dataset Compounds (56 compounds) and designed CD73 inhibitors (52 compounds).

**CD73 Dataset Compounds**	
Compound	SMILES Structure
1	OC1=CC=CC(C2=NN=C3N2C=C(C4=COC=C4)C=C3)=C1
2	OC1=CC=CC(C2=NNC3=C2C=C(C4=COC=C4)C=C3)=C1
3	OC1=CC=CC(N2C=NC3=C2N=C(C4=COC=C4)N=C3)=C1
4	OC1=CC=CC(N2N=NC3=C2C=C(C4=COC=C4)C=C3)=C1
5	OC1=CC=CC(C2=NN=C3N2C=C(C4=COC=C4)C=C3)=C1F
6	C12=NN=C(C3=CN=CC=C3)N1C=C(C4=COC=C4)C=C2
7	O=C(C1)NC2=C1C=CC(C3=NN=C4N3C=C(C5=COC=C5)C=C4)=C2
8	C12=NN=C(C3=CC(NN=C4)=C4C=C3)N1C=C(C5=COC=C5)C=C2
9	OC1=CC=CC(C2=NN=C3N2C=C(C4=C(C)OC=C4)C=C3)=C1
10	OC1=CC=CC(C2=NN=C3N2C=C(C4=CC=NN4C)C=C3)=C1
11	OC1=CC=CC(C2=NN=C3N2C=C(C4=CN=NN4)C=C3)=C1
12	OC1=CC=CC(C2=NN=C3N2C=C(C4=CNN=C4)C=C3)=C1
13	OC1=CC=CC(C2=NN=C3N2C=C(C4=CON=C4)C=C3)=C1
14	OC1=CC=CC(C2=NN=C3N2C=C(N4C=CC=N4)C=C3)=C1
15	OC1=CC=CC(C2=NN=C3N2C=C(C4=CC=NN4)C=C3)=C1
16	OC1=CC=CC(C2=NN=C3N2C=C(C4=CC=CN4)C=C3)=C1
17	OC1=CC=CC(C2=NN=C3N2C=C(C4=CC=NS4)C=C3)=C1
18	OC1=CC=CC(N2N=NC3=C2C=C(C4=CC=NN4C)C=C3)=C1
19	OC1=CC=CC(N2N=NC3=C2C=C(C4=CC=NN4C)C=C3NCCO)=C1
20	OC1=CC=CC(N2N=NC3=C2C=C(C4=CC=NN4C)C=C3Cl)=C1

21	OC1=CC=CC(N2N=NC3=C2C=C(C4=CC=NN4C)C=C3C#N)=C1
22	OC1=CC=CC(N2N=NC3=C2C=C(C4=CC=NN4C)C=C3C)=C1
23	OC1=CC=CC(N2N=NC3=C2C=C(C4=CC=NN4C)C=C3C=C)=C1
24	OC1=CC=CC(N2N=NC3=C2C=C(C4=CC=NN4C)C=C3CC)=C1
25	C1(N=NN2C3=CC(NN=C4)=C4C=C3)=C2C=C(C5=CC=NN5C6=CC=CC=C6)C=C1
26	CC1=CC=CC(N2N=CC=C2C3=CC4=C(N=NN4C5=CC(NN=C6)=C6C=C5)C=C3)=C1
27	CC1=CC=CC(N2N=CC=C2C3=CC4=C(N=NN4C5=CC(NN=C6)=C6C=C5)C(F)=C3)=C1
28	CC1=CC=CC(N2N=CC=C2C3=CC4=C(N=NN4C5=CC(NN=C6)=C6C=C5)C(Cl)=C3)=C1
29	N#CC1=CC=CC(N2N=CC=C2C3=CC4=C(N=NN4C5=CC(NN=C6)=C6C=C5)C=C3)=C1
30	FC(C1=CC=CC(N2N=CC=C2C3=CC4=C(N=NN4C5=CC(NN=C6)=C6C=C5)C=C3)=C1)(F)F
31	COC1=CC=CC(N2N=CC=C2C3=CC4=C(N=NN4C5=CC(NN=C6)=C6C=C5)C=C3)=C1
32	FC(OC1=CC=CC(N2N=CC=C2C3=CC4=C(N=NN4C5=CC(NN=C6)=C6C=C5)C=C3)=C1)(F)F
33	FC1=CC=CC(N2N=CC=C2C3=CC4=C(N=NN4C5=CC(NN=C6)=C6C=C5)C=C3)=C1
34	ClC1=CC=CC(N2N=CC=C2C3=CC4=C(N=NN4C5=CC(NN=C6)=C6C=C5)C=C3)=C1
35	C1(N=NN2C3=CC(NN=C4)=C4C=C3)=C2C=C(C5=CC=NN5CC6=CC=CC=C6)C=C1
36	FC1=CC(C2=CC=NN2CC3=CC=CC=C3)=CC4=C1N=NN4C5=CC(NN=C6)=C6C=C5
37	ClC1=CC(C2=CC=NN2CC3=CC=CC=C3)=CC4=C1N=NN4C5=CC(NN=C6)=C6C=C5
38	ClC1=CC=CC=C1CN2N=CC=C2C3=CC4=C(N=NN4C5=CC(NN=C6)=C6C=C5)C=C3
39	ClC1=CC(CN2N=CC=C2C3=CC4=C(N=NN4C5=CC(NN=C6)=C6C=C5)C=C3)=CC=C1
40	ClC(C=C1)=CC=C1CN2N=CC=C2C3=CC4=C(N=NN4C5=CC(NN=C6)=C6C=C5)C=C3
41	CC1=CC=CC=C1CN2N=CC=C2C3=CC4=C(N=NN4C5=CC(NN=C6)=C6C=C5)C=C3
42	CC(C=C1)=CC=C1CN2N=CC=C2C3=CC4=C(N=NN4C5=CC(NN=C6)=C6C=C5)C=C3
43	C1(N=NN2C3=CC(NN=C4)=C4C=C3)=C2C=C(C5=CC=NN5CC6=NC=CC=C6)C=C1
44	C1(N=NN2C3=CC(NN=C4)=C4C=C3)=C2C=C(C5=CC=NN5CC6=CN=CC=C6)C=C1
45	C1(N=NN2C3=CC(NN=C4)=C4C=C3)=C2C=C(C5=CC=NN5CC6=CC=NC=C6)C=C1
46	O=S(C(C=C1)=CC=C1CN2N=CC=C2C3=CC4=C(N=NN4C5=CC(NN=C6)=C6C=C5)C=C3)(C)=O
47	CCCC(C=C1)=CC=C1CN2N=CC=C2C3=CC4=C(N=NN4C5=CC(NN=C6)=C6C=C5)C=C3
48	N#CC(C=C1)=CC=C1CN2N=CC=C2C3=CC4=C(N=NN4C5=CC(NN=C6)=C6C=C5)C=C3
49	FC1=CC(C2=CC=NN2CC3=CC=C(C#N)C=C3)=CC4=C1N=NN4C5=CC(NN=C6)=C6C=C5
50	ClC1=CC(C2=CC=NN2CC3=CC=C(C#N)C=C3)=CC4=C1N=NN4C5=CC(NN=C6)=C6C=C5
51	ClC(C=C1Cl)=CN=C1CN2N=CC=C2C3=CC4=C(N=NN4C5=CC(NN=C6)=C6C=C5)C=C3
52	ClC(C=C1)=CN=C1CN2N=CC=C2C3=CC4=C(N=NN4C5=CC(NN=C6)=C6C=C5)C=C3
53	ClC1=CC=CN=C1CN2N=CC=C2C3=CC4=C(N=NN4C5=CC(NN=C6)=C6C=C5)C=C3
54	N#CC(C=C1)=CN=C1CN2N=CC=C2C3=CC4=C(N=NN4C5=CC(NN=C6)=C6C=C5)C=C3
55	FC1=CC(Cl)=CC=C1CN2N=CC=C2C3=CC4=C(N=NN4C5=CC(NN=C6)=C6C=C5)C=C3
56	ClC1=CC(Cl)=CC=C1CN2N=CC=C2C3=CC4=C(N=NN4C5=CC(NN=C6)=C6C=C5)C=C3

CD73 Designed Compounds	
D1	CC1=NC(C2=CC=NN2CC3=CC=C(C#N)C=C3)=NC4=C1N=CC(C5=CC(NN=C6)=C6C=C5)=C4
D2	OC1=CC=CC(C2=C3C(N=C2)=COC(C4=C(CC5=CC(Cl)=C(S(=O)(C(F)(F)F)=O)C=C5)N=NN4)=C3)=C1
D3	N#CC(C=C1)=CC=C1CC2=C(SC=N2)C3=CC4=C(C5=CC(NN=C6)=C6C=C5)N=NC4=CO3
D4	N#CC(C=C1)=CC=C1CN2N=CC=C2C3=CC4=C(N=NN4C5=CC(NCS6)=C6C=C5)C=C3
D5	CC(SCC1)NC1N2N=NC3=C2C=C(C4=CC=NN4CC5=CC=C(C#N)C=C5)C=C3
D6	OC1=NC=CC(N2N=NC3=C2C=C(C4=CC=NN4CC5=CC=C(C#N)C=C5)C=C3)=N1
D7	OC1=CC=CC(N2N=NC3=C2C=C(C4=CC=NN4CC5=CC=C(C#N)C=C5)C=C3)=C1
D8	O=S(C(C=C1)=CC=C1CN2N=CC=C2C3=CC4=C(N=NN4C5=CC(NN=C6)=C6C=C5)C=C3)(C(F)(F)F)=O
D9	FC(C(C=C1)=C(C(F)(F)F)C=C1CN2N=CC=C2C3=CC4=C(N=NN4C5=CC(NN=C6)=C6C=C5)C=C3)(F)F
D10	ClC1=C(S(=O)(C(F)(F)F)=O)C=CC(CN2N=CC=C2C3=CC4=C(N=NN4C5=CC(NN=C6)=C6C=C5)C=C3)=C1
D11	ClC(C=C(S(=O)(C(F)(F)F)=O)C=C1)=C1CN2N=CC=C2C3=CC4=C(N=NN4C5=CC(NN=C6)=C6C=C5)C=C3
D12	BrC1=C(S(=O)(C(F)(F)F)=O)C=CC(CN2N=CC=C2C3=CC4=C(N=NN4C5=CC(NN=C6)=C6C=C5)C=C3)=C1
D13	BrC(C=C(S(=O)(C(F)(F)F)=O)C=C1)=C1CN2N=CC=C2C3=CC4=C(N=NN4C5=CC(NN=C6)=C6C=C5)C=C3
D14	N#CC(C=C1)=CC=C1CC2=NOC=C2C3=CC4=C(N=NN4C5=CC(NN=C6)=C6C=C5)C=C3
D15	N#CC(C=C1)=CC=C1CC2=C(NN=N2)C3=CC4=C(N=NN4C5=CC(NN=C6)=C6C=C5)C=C3
D16	N#CC(C=C1)=CC=C1CC2=NSN=C2C3=CC4=C(N=NN4C5=CC(NN=C6)=C6C=C5)C=C3
D17	N#CC(C=C1)=CC=C1CC2=CC(C3=CC4=C(N=NN4C5=CC(NN=C6)=C6C=C5)C=C3)=CC=N2
D18	CC1=NC(C2=CC=NN2CC3=CC(Cl)=C(S(=O)(C(F)(F)F)=O)C=C3)=NC4=C1N=CC(C5=CC(NN=C6)=C6C=C5)=C4
D19	CC1=NC(C2=CC=NN2CC3=C(Cl)C=C(S(=O)(C(F)(F)F)=O)C=C3)=NC4=C1N=CC(C5=CC(NN=C6)=C6C=C5)=C4
D20	CC1=NC(C2=CC=NN2CC3=CC(Br)=C(S(=O)(C(F)(F)F)=O)C=C3)=NC4=C1N=CC(C5=CC(NN=C6)=C6C=C5)=C4
D21	CC1=NC(C2=CC=NN2CC3=C(Br)C=C(S(=O)(C(F)(F)F)=O)C=C3)=NC4=C1N=CC(C5=CC(NN=C6)=C6C=C5)=C4
D22	ClC1=C(S(=O)(C(F)(F)F)=O)C=CC(CN2N=CC=C2C3=CC4=C(C5=CC(NN=C6)=C6C=C5)N=NC4=CO3)=C1
D23	ClC(C=C(S(=O)(C(F)(F)F)=O)C=C1)=C1CN2N=CC=C2C3=CC4=C(C5=CC(NN=C6)=C6C=C5)N=NC4=CO3
D24	BrC1=C(S(=O)(C(F)(F)F)=O)C=CC(CN2N=CC=C2C3=CC4=C(C5=CC(NN=C6)=C6C=C5)N=NC4=CO3)=C1
D25	BrC(C=C(S(=O)(C(F)(F)F)=O)C=C1)=C1CN2N=CC=C2C3=CC4=C(C5=CC(NN=C6)=C6C=C5)N=NC4=CO3
D26	BrC(C=C(S(=O)(C(F)(F)F)=O)C=C1)=C1CC2=NOC=C2C3=CC4=C(C5=CC(O)=CC=C5)N=NC4=CO3
D27	CC1=NC(C2=C(CC3=CC(Cl)=C(S(=O)(C(F)(F)F)=O)C=C3)N=NN2)=NC4=C1N=CC(C5=CC(O)=CC=C5)=C4
D28	CC1=NC(C2=NSN=C2CC3=CC(Cl)=C(S(=O)(C(F)(F)F)=O)C=C3)=NC4=C1N=CC(C5=CC(O)=CC=C5)=C4
D29	CC1=NC(C2=CC=NC(CC3=CC(Cl)=C(S(=O)(C(F)(F)F)=O)C=C3)=C2)=NC4=C1N=CC(C5=CC(O)=CC=C5)=C4

D30	CC1=NC(C2=NSN=C2CC3=C(Cl)C=C(S(=O)(C(F)(F)F)=O)C=C3)=NC4=C1N=CC(C5=CC(O)=CC=C5)=C4
D31	CC1=NC(C2=CC=NC(CC3=C(Cl)C=C(S(=O)(C(F)(F)F)=O)C=C3)=C2)=NC4=C1N=CC(C5=CC(O)=CC=C5)=C4
D32	CC1=NC(C2=CON=C2CC3=CC(Br)=C(S(=O)(C(F)(F)F)=O)C=C3)=NC4=C1N=CC(C5=CC(O)=CC=C5)=C4
D33	CC1=NC(C2=C(CC3=CC(Br)=C(S(=O)(C(F)(F)F)=O)C=C3)N=NN2)=NC4=C1N=CC(C5=CC(O)=CC=C5)=C4
D34	CC1=NC(C2=NSN=C2CC3=CC(Br)=C(S(=O)(C(F)(F)F)=O)C=C3)=NC4=C1N=CC(C5=CC(O)=CC=C5)=C4
D35	CC1=NC(C2=C(CC3=C(Br)C=C(S(=O)(C(F)(F)F)=O)C=C3)N=NN2)=NC4=C1N=CC(C5=CC(O)=CC=C5)=C4
D36	CC1=NC(C2=NSN=C2CC3=C(Br)C=C(S(=O)(C(F)(F)F)=O)C=C3)=NC4=C1N=CC(C5=CC(O)=CC=C5)=C4
D37	OC1=CC=CC(C2=C3C(N=N2)=COC(C4=CON=C4CC5=CC(Cl)=C(S(=O)(C(F)(F)F)=O)C=C5)=C3)=C1
D38	OC1=CC=CC(C2=C3C(N=N2)=COC(C4=C(CC5=CC(Cl)=C(S(=O)(C(F)(F)F)=O)C=C5)N=NN4)=C3)=C1
D39	OC1=CC=CC(C2=C3C(N=N2)=COC(C4=NSN=C4CC5=CC(Cl)=C(S(=O)(C(F)(F)F)=O)C=C5)=C3)=C1
D40	OC1=CC=CC(C2=C3C(N=N2)=COC(C4=CC=NC(CC5=CC(Cl)=C(S(=O)(C(F)(F)F)=O)C=C5)=C4)=C3)=C1
D41	OC1=CC=CC(C2=C3C(N=N2)=COC(C4=CON=C4CC5=C(Cl)C=C(S(=O)(C(F)(F)F)=O)C=C5)=C3)=C1
D42	OC1=CC=CC(C2=C3C(N=N2)=COC(C4=C(CC5=C(Cl)C=C(S(=O)(C(F)(F)F)=O)C=C5)N=NN4)=C3)=C1
D43	OC1=CC=CC(C2=C3C(N=N2)=COC(C4=NSN=C4CC5=C(Cl)C=C(S(=O)(C(F)(F)F)=O)C=C5)=C3)=C1
D44	OC1=CC=CC(C2=C3C(N=N2)=COC(C4=CC=NC(CC5=C(Cl)C=C(S(=O)(C(F)(F)F)=O)C=C5)=C4)=C3)=C1
D45	OC1=CC=CC(C2=C3C(N=N2)=COC(C4=CON=C4CC5=CC(Br)=C(S(=O)(C(F)(F)F)=O)C=C5)=C3)=C1
D46	OC1=CC=CC(C2=C3C(N=N2)=COC(C4=C(CC5=CC(Br)=C(S(=O)(C(F)(F)F)=O)C=C5)N=NN4)=C3)=C1
D47	OC1=CC=CC(C2=C3C(N=N2)=COC(C4=NSN=C4CC5=CC(Br)=C(S(=O)(C(F)(F)F)=O)C=C5)=C3)=C1
D48	OC1=CC=CC(C2=C3C(N=N2)=COC(C4=CC=NC(CC5=CC(Br)=C(S(=O)(C(F)(F)F)=O)C=C5)=C4)=C3)=C1
D49	BrC(C=C(S(=O)(C(F)(F)F)=O)C=C1)=C1CC2=NOC=C2C3=CC4=C(C5=CC(O)=CC=C5)N=NC4=CO3
D50	BrC(C=C(S(=O)(C(F)(F)F)=O)C=C1)=C1CC2=C(NN=N2)C3=CC4=C(C5=CC(O)=CC=C5)N=NC4=CO3
D51	BrC(C=C(S(=O)(C(F)(F)F)=O)C=C1)=C1CC2=NSN=C2C3=CC4=C(C5=CC(O)=CC=C5)N=NC4=CO3
D52	BrC(C=C(S(=O)(C(F)(F)F)=O)C=C1)=C1CC2=CC(C3=CC4=C(C5=CC(O)=CC=C5)N=NC4=CO3)=CC=N2

The SMILES code for CD73 Dataset compounds are related to Table S1 and SMILES code for CD73 Designed compounds are related to Table S2.

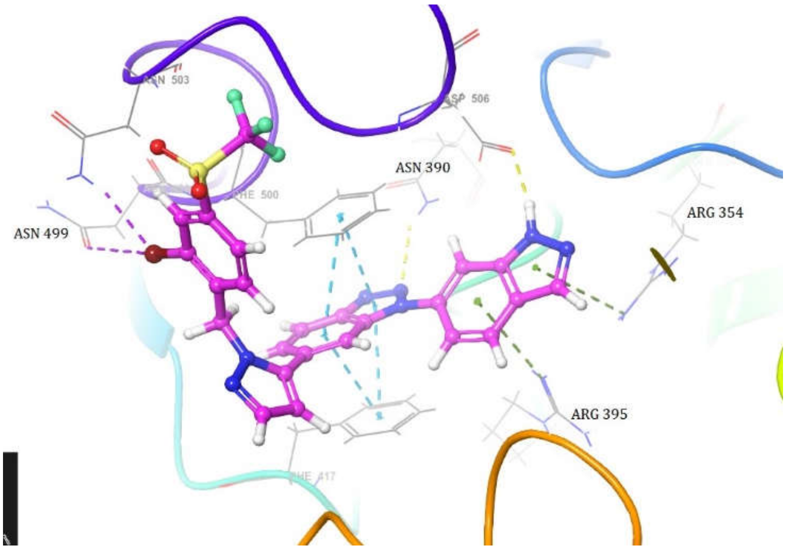

Figure S1: The representative docked pose of the most potent designed CD74 inhibitor (D13) inside the active site of CD73.

(Hydrogen bonds are represented as yellow dotted lines, pi-pi interactions are represented as cyan dotted lines, and pi-cation interactions are represented as green dotted lines, halogen bonds are represented as purple dotted lines).
